# Molecular allergy diagnostic tests: development and relevance in clinical practice 

**DOI:** 10.5414/ALX01617E

**Published:** 2017-08-04

**Authors:** J. Kleine-Tebbe, U. Jappe

**Affiliations:** 1 Allergie- und Asthma-Zentrum Westend, Praxis Hanf, Ackermann und Kleine-Tebbe, Berlin,; 2 Forschungsgruppe Klinische und Molekulare Allergologie, Forschungszentrum Borstel, Deutsches Zentrum für Lungenforschung, and; 3 Klinik für Dermatologie, Allergologie und Venerologie, Universität zu Lübeck, Germany

**Keywords:** molecular allergy, single allergen, allergen component, allergy diagnostics, IgE, IgE test

## Abstract

Molecular allergy is based on identification, characterization and subsequent use of single allergens, being components of complex allergen sources like pollen, mites, furred animals, foods or insect venoms. Only few protein families contain relevant allergens of similar sequence and structure, carrying common IgE epitopes as the basis of cross reactivity. Used as purified or recombinant (glyco)proteins single allergens can potentially improve in-vitro diagnostics, particularly allergen-specific IgE assays through a) increased sensitivity, b) use of risk and marker allergens, c) component-resolved diagnostics (CRD). CRD can differentiate primary, species-specific from secondary, cross-reactive sensitizations to single allergens. Allergen components facilitate an increased analytical sensitivity, particularly if they are underrepresented or missing in conventional allergen extracts. They are mainly used in single assays (singleplex) for the detection of IgE, but also in a microarray format (multiplex) with 112 components from 50 allergen sources with slightly decreased analytical sensitivity. Concepts of molecular allergy can only be separately defined and utilized for each allergen source (pollen, mites, foods or insect venoms). As soon as essential singe allergens are available, their specific role in diagnostics should be defined. This requires well characterized patient cohorts from various countries, since exposure, allergic immune response and clinical relevance can vary substantially between individual subjects and geographical regions. The patient’s clinical information is essential for proper interpretation of molecular allergology results. The history and/or challenge test results will finally provide evidence, in how far a sensitization to single allergens might be clinically relevant or not.

German version published in Allergologie, Vol. 36, No. 8/2013, pp. 327-349

## Introduction 

In the past decades, numerous single allergens have been identified and described; some of them are available for clinical investigation and routine diagnostic work-up. Certain single allergens improve diagnosis, in others the corresponding specific IgE response is redundant or clinically irrelevant. Therefore, the advantages of molecular allergy investigation cannot be evaluated as a whole, but have to be defined individually for each allergen source and each single allergen. 

In this review, we report the challenges of the development and the concepts of molecular allergy diagnosis in clinical practice. The use of single allergens does not only provide potential advantages for the development of new preparations and for investigation (Text box 1), but also for the diagnostic work-up in allergy patients. 

## Production, denomination, and classification of single allergens 

### Results obtained in 50 years of allergen research

As early as the 1960s, a rapidly increasing understanding of immunology and a growing interest in the allergens responsible for IgE-mediated immune reactions lead scientific pioneers to purify and use protein allergens (including ragweed major allergen Amb a 1, formerly antigen E) for immunotherapy [1]. In the 1970s and 80s, single allergens like Amb a 5 were used to detect HLA associations in specific sensitizations [2]. Although human IgE responses to major allergens are hardly associated with MHC class II alleles, the first major allergens identified for this purpose have been a driving force in the investigation of protein allergens with recombinant technology (review and historical development in [3]). 

In the meantime, more than 1,000 protein allergens have been identified (www.allergome.org), characterized, and – most of them – officially denominated (www.allergen.org). Innovative techniques in molecular biology have significantly accelerated this process. Nevertheless, the task of producing a recombinant protein that is folded identically to its natural counterpart still remains difficult and challenging, even more so when the protein’s structure is highly complex. 

### Isoforms: natural allergen variants

In nature, allergens are present in various variations (isoforms), which can differ in only a few amino acids or in complete peptide segments. These deviations of the primary structure (amino acid sequence) can have an effect on IgE binding due to a different folding and structure of the protein. When deciding on a certain allergen molecule, it is therefore important to consider whether the chosen representative is recognized by all allergen-specific IgE antibodies. This problem is not present when natural single allergens that represent a mixture of various isoforms and can additionally have carbohydrate side chains are used. The latter can be important but can also interfere in IgE binding. 

In contrast, recombinant allergens produced in bacteria do not have glucose side chains, which can be an advantage but also a disadvantage for the folding of the entire molecule. 

**Figure 1.**
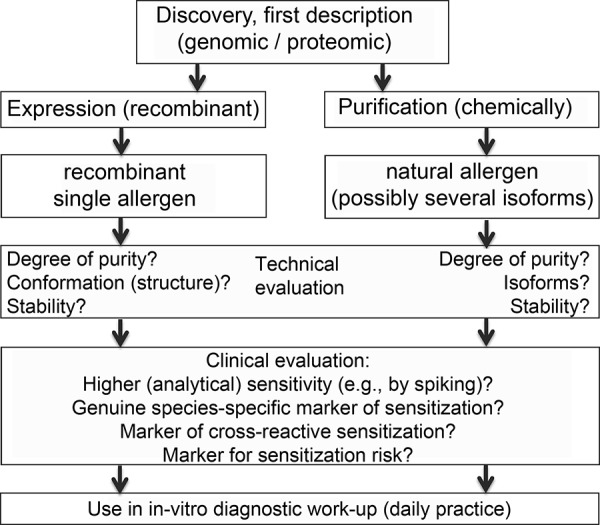
Development of allergen molecules for diagnostic work-up.

**Figure 2.**
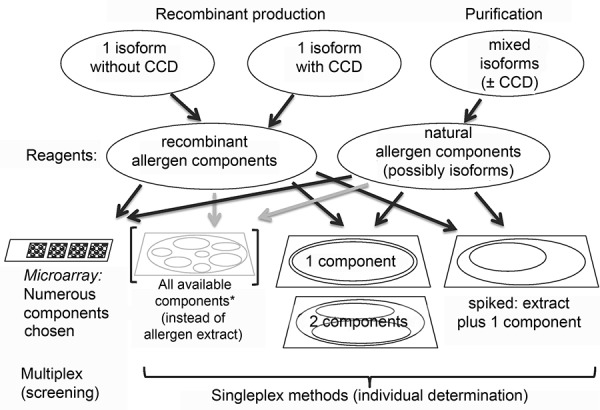
Selection and use of allergen molecules for IgE diagnosis. CCD = cross-reactive carbohydrate determinants.

This illustrates that many challenges have to be mastered along the way from the discovery of allergen molecules to their clinical use (Figure 1, Figure 2). 

### Protein families and nomenclature

In the past decades, the coding genes of many organisms as well as the corresponding protein structures (from the primary to – in some cases – the tertiary structure) have been determined. Open-access databases facilitate the search for related genes, peptides, proteins, and molecules with similar structures. The latter allow researchers to sort proteins according to families of which only a few incorporate the known allergens [4] (www.meduniwien.ac.at/allergens/allfam/), for instance:

- The Bet v 1 superfamily,
- cupin superfamily (including storage proteins: vicilins, legumins),
- the prolamin superfamily (including storage proteins: 2S albumins, non-specific lipid transfer proteins LTP),
- the profilins,
- the Ca^++^-binding proteins (proteins with EF-hand motif),
- the lipocalins.

Cross-reactive carbohydrate determinants (CCD) as additional side chains on the surface of glycoproteins play a special role. 

The official allergen nomenclature for single allergens [5, 6, 7] is based on:

- the abbreviated Latin name of the allergen source (the first three letters of the genus),
- the first letter of the species name, and
- the number of the allergen in sequence of discovery (if a homologous allergen is known from another species, its allergen number is taken if possible).

For example, Bet v 1 is the name of the major allergen of the (silver) birch Betula verrucosa. Newly discovered single allergens are submitted to the nomenclature subcommittee of the *International Union of Immunological Societies *(IUIS); when sufficient scientific documentation is available, they are accepted by this committee, given an official name [7], and registered (www.allergen.org). 

## Sources of information on single allergens and allergen families 

Information on molecular allergy can be obtained from various sources: 

In *standard works* and *textbooks* on allergy, basic knowledge of molecular biology is provided (see e.g., Chapter 9 in [8]). Nevertheless, the practical use of single allergens in routine diagnosis is neglected. 

*Review articles on molecular allergy* [9, 10, 11 – series in Allergo Journal since 2010] are also suitable to keep the interested reader up-to-date. The number of original publications in the field of molecular allergy research has also been increasing lately, which makes it more difficult to gain an overview of the entire topic. 

In addition, *open-access allergen databases (*e.g., www.allergome.org) have done real pioneering work. The information on allergen molecules provided, the linking to existing scientific databases as well as the biographical references, have made these databases an indispensable tool (Table 1). 

Regular updating ensures up-to-date information from predominantly newly published original papers. 

Furthermore, manufacturers of recombinant allergens provide *online information on allergen molecules* and their possible use. Over the past years, more and more courses and web seminars are becoming available (http://www.allergyeducation-ma.com). 

## Potential use of single allergens 

### Characterization and standardization of allergen extracts

It is only through the knowledge of single allergens that the characterization and standardization of allergen extracts becomes possible. The identification of causative allergens in the extracts of pollen, mites, or animal dander, for example, allows researchers to optimize and compare these products. Currently, two major allergens (Bet v 1 from birch pollen; Phl p 5a from timothy grass pollen) are offered as reference preparations (http://crs.edqm.eu/db/) by the European Directorate for the Quality of Medicines and Health Care (EDQM, http://www.edqm.eu). After the first ring tests have been completed, additional monoclonal antibody pairs will soon make it possible to establish standardized and comparable immune tests for the determination of the two (intact) major allergens in allergen products or their precursors. With this, the declaration of major allergens contained in products for specific immunotherapy would be achieved for the first time – a dream come true for allergists. 

### Determination of allergens and environmental monitoring

The determination of allergens in the environment is a new domain where allergen molecules and their specific monoclonal antibodies can be used. Immune tests with single allergens as a reference allow for the indoor (http://www.inbio.com) and outdoor measurement of certain allergens. Furthermore, the food industry has also developed sensitive test methods for the detection of certain allergens; these methods are frequently based on the knowledge and availability of the causative single allergens [12]. 

### Preparations for specific immunotherapy

Manufacturers of allergens are developing therapeutic preparations based on single allergens for specific immunotherapy [13]. They are currently being clinically evaluated and will soon be available for specific immunotherapy of tree and grass pollen allergies. 

## Diagnostic testing with single allergens 

### In-vivo diagnostic testing with single allergens

Allergen molecules have been used for diagnostic testing since they were discovered and became available. *Titrated skin tests with natural or recombinant allergens *[14, 15] showed dose-dependent immediate-type reactions at concentrations in the microgram range (µg/mL). Certain reagents were also used in *nasal or bronchial challenge testing *[15, 16]. The pioneer papers have confirmed the concept of molecular allergy diagnosis with the available devices for in-vivo testing. 

Nevertheless, *recombinant allergens* will not be available for routine in-vivo diagnostic work-up in the near future. The most important reason for this is the significant effort involved in scientific and clinical documentation in order to achieve marketing authorization for these preparations. The costs of these technical and clinical validations would by far exceed the expected profit. 

Another situation is encountered in the case of* naturally purified allergen molecules,* which can be more easily prepared for marketing authorization as diagnostic reagents. 

### Potential reagents for prick testing

There are interesting reagents based on pollen pan allergens that are already being used by Spanish allergists for prick testing. These pollen extracts stem from date palms and do not contain any known tree pollen major allergens. The purified profilin fraction can be used for diagnosis of patients sensitized to profilin. The remainder of the fraction from these extracts contains other cross-reactive pan allergens, e.g., polcalcin, a Ca^++^-binding protein, which is present in all pollen species. In Spain, these reagents have proven effective [17] in identifying patients with assumed “multi allergy” as being pan pollen allergic and in obtaining hints towards sensitizations against profilin or polcalcin (Table 2), see also [18]. 

It would be desirable that manufacturers of allergens (currently ALK-Abelló, LETI, Spain) develop these reagents for other European regions as well in order to establish a simple screening method for patients with pan-pollen allergy (in Germany about 10 – 15% of patients with pollen allergy). 

## Determination of specific IgE antibodies against single allergens 

### Progress in-vitro diagnosis

For daily practice, the biggest advance of molecular allergy has been made in in-vitro diagnosis. Initially, in-vitro diagnosis was only possible with a small selection of naturally purified allergens (e.g., derived from cow’s milk); today, a large spectrum of predominantly recombinant single allergens is available. They are used – independently of the assay system applied – in the same way as allergen extracts and either bind to the corresponding matrix in solid phase systems (activated nitrocellulose, cellulose polymers with a large surface, polystyrene plastic surfaces) or to other commercial media (Table 3). 

### Comparability of specific IgE values

Even when single allergens are used, it is difficult to compare the allergen-specific IgE concentrations in the products of different manufacturers due to several differences:

- plate systems,
- purification methods,
- protocols for the recombinant production of allergens,
- procedures for the bonding of single allergens,
- detection systems and evaluation protocols.

These limitations are similar to that of the use of allergen extracts for determination of specific IgE. When single allergens are used, it can be expected that the differences between the results of different manufacturers are smaller than when allergen extracts are used for the determination of specific IgE; adequate comparative trials are not yet available. 

### IgE testing for individual detection or mass screening

Allergen-specific IgE antibodies can be detected individually (*singleplex)* using various systems that have different levels of automation (Table 3). Screening tests based on modern microchip technology allow for the parallel measurement *(multiplex)* of IgE from a small serum sample (20 µl) against, currently, 112 allergen components (ImmunoCAP ISAC, ThermoFisher, Freiburg, Germany). 

The two methods that are most frequently used for in-vitro diagnosis of allergy in German-speaking countries are a solid-phase system (ImmunoCAP, formerly Phadia, now ThermoFisher, Freiburg, Germany) and a liquid allergen system on the laboratory platform Immulite 2000 (Siemens Health Care Diagnostics GmbH, Eschborn, Germany). Other manufacturers also offer mainly naturally purified single allergens for allergy diagnosis (Table 3). 

### Evaluation of IgE determination using components

The available data on in-vitro diagnosis with single allergens are mainly related to the ImmunoCAP and the ISAC system. Numerous publications have evaluated diagnostic parameters and clinical suitability for certain allergen sources, mainly food (selection of important original papers are presented in Table 4). Less published data are available on the usefulness of single allergens produced by other manufacturers so that currently neither the technical nor the clinical usefulness of all components can be assessed. 

Inclusion criteria in clinical trials (e.g., proof of sensitization by extract diagnosis) can be another problem, especially when the extracts used lack important single allergens [19]; for instance, how can the single allergens of peanut be validated comprehensively if some patients with peanut allergy are not included in a trial due to inadequate peanut extracts? This example illustrates the problem of under-represented or missing single allergens in certain extracts. Only the combination of various extraction methods could make it possible to warrant the presence of all relevant single allergens [19]. 

The manufacturers of procedures for IgE diagnosis are recommended to document at least the technical but if possible also the clinical usefulness of the new reagents as soon as the single allergens for specific IgE determination are introduced on the market. 

### Lack of regulation and consequences

In the USA, unlike in Europe, the allergen reagents used are regulated by the Food and Drug Administration (FDA). 

The necessary procedures for marketing authorization have led to a delayed market launch of single allergens for in-vitro diagnosis there. Currently, only a few single food allergens are registered (“cleared”), but the wholesale laboratories do not yet offer them for end-users. The comparison illustrates that, in Europe, the missing regulations for in-vitro allergen reagents facilitate their quick market launch, but at the price of a partially inadequate documentation of their diagnostic usefulness. The same is true for all allergen extracts for in-vitro diagnosis. 

## Fundamental capabilities of components for diagnostic work-up 

### Component-specific diagnostic work-up and spiking of extracts

With the help of single allergens, serologic in-vitro diagnosis can be modified in several ways (Figure 2): 

A) Components or single allergens can be used individually as a reagent for determination of specific IgE (currently, the most common use). 

B) Single components can be added to allergen extracts (“spiking”) in order to increase the sensitivity of the test (e.g., in the case of under-represented components). 

C) All available single components of an allergen source could be used as a mixture instead of a complex allergen extract (this is theoretically possible, but has not been done so far because it is complex, expensive, and of questionable use). 

Option A allows a strict differentiation of sensitizations according to allergen components. This procedure has been called Component Resolved Diagnostic (CRD) [20] and currently plays the most important role for molecular allergy. 

Improving the allergen extracts by specific spiking (Option B) provides significant benefit for the diagnosis of latex allergy: Since the under-represented latex allergen Hev b 6 has been added to the extract (ImmunoCAP k82) [21], the sensitivity has markedly improved and corresponds to that of a mixture of all available single allergens. Another example is hazel extract (ImmunoCAP f17), which has been spiked with the Bet v 1 cross-reactive hazel allergen Cor a 1. 

However, this spiked hazel extract does not only detect IgE-mediated sensitization against stable storage proteins (major part) or other components (e.g., lipid transfer protein) of the extract but also (partially clinically irrelevant) cross-reactions in tree pollen allergy. This can be a problem, for example for US pediatricians who used to use the extract mainly for the diagnosis of hazel allergy in infants and children (usually against storage proteins). 

This illustrates that spiking can improve test sensitivity (e.g., for Bet v 1-homologous food allergy), but at the same time can lead to a number of unwelcome results in the diagnosis of allergy to storage proteins. 

### Differentiation of purified and recombinant components

A very important decision for manufacturers is whether to use naturally purified components in all their variations (isoforms) or to choose a single, recombinantly produced protein. 

The latter should be representative and contain the most important IgE binding sites to be able to detect all patients sensitized to this component. This is not a problem when natural components are used; however, the natural components must be free of impurities and can “falsify” the result due to additional sugar bindings when the subject possesses carbohydrate-specific IgE antibodies. 

By choosing the appropriate expression system, manufacturers can use recombinant components to produce the protein without (e.g., in E. coli bacteria) or with carbohydrate side chains (e.g., insect or yeast cells). 

Some manufacturers, however, are not allowed to use recombinant components due to patent protection and have to purify the allergen components. This is more laborious but results in “nature-identical” allergens (Table 1). 

## Multiplex procedure (microarray) for diagnostic work-up using components 

### Design and operating mode of a microarray IgE test

Newly developed microarray technologies allow for the miniaturization of any binding assay, among them also immunoassays. A microarray for the semi-quantitative determination of allergen-specific IgE antibodies against single allergens from more than 50 airborne and food allergens is already available. 

In this multiplex procedure (ImmunoCAP ISAC) 112 immobilized allergen components are attached to a glass slide: 

1. The antibodies in the patient serum bind to the bonded allergen components. 

2. After a washing step, the allergen-specific antibodies are detected using a fluorescence-marked anti-IgE antibody. 

3. The test results are measured using a laser scanner and interpreted using PC software. 

The semi-quantitative test results are indicated in *ISAC Standardized Units (ISU).* Due to the large range of allergen components used (4 serum zones per glass slide / biochip), 112 tests are carried out at the same time, the (parallel) evaluation of which is difficult. 

### Technical evaluation

Some (technical as well as clinical) results of external evaluations have already been published [22, 23]. 

Data of the in-company evaluation are available for most components (data on file, available upon request, ThermoFisher, Freiburg, Germany) and are related to several basic parameters of commercially available immunoassays:

- precision (reproducibility of results depending on the signal strength),
- intra-assay variation coefficient (deviations within a test run),
- inter-assay variation coefficient (deviation between different test runs),
- linearity (measurement characteristics of serial solutions),
- detection limit (upper and lower),
- matrix aspects (influence of extreme serum conditions, lipids, hemolysis),
- total IgE interference (influence of extremely high IgE concentrations),
- comparison with singleplex measurements (e.g., ImmunoCAP single determinations).

The optimization of this microarray system for the determination of numerous component-specific IgE antibodies has to be considered a continuous process. In the future, for example, more adequate components, a different choice, special panels (grouped allergens for diagnosis), interpretation aids (Xplain, http://www.phadia.com/da/Products/Software-Services/ImmunoCAP-ISAC-Explain/), and automation might improve the system. 

### Fields of application, potential advantages and challenges

Microarray IgE tests can be applied in the following clinical settings:

- multiple and complex patterns of sensitization against inhalant allergens (pollen, or also animal dander and mites),
- numerous pollen sensitizations with the corresponding associated cross-reactions against vegetable food,
- unclear food reactions (also without pollen allergy) against various foods,
- unclear triggers of anaphylaxis when IgE-mediated immediate-type reaction is suspected.

The clear advantage of microarray tests lies in the high number of allergen components that can be tested in a low amount of serum (20 µl). Thanks to this, one test run suffices to detect several sensitizations that can provide a comprehensive insight into the patient’s possible allergen spectrum. As the clinical relevance of each single one of these sensitizations has to be clarified, certain problems may arise: 

A. When the total IgE is markedly increased – which frequently occurs in patients with atopic eczema – the high number of reactions to various components can be challenging. This is particularly true for components from allergen sources for which a challenge test for clinical relevance is not possible. 

B. Unexpected and, due to a negative patient history, irrelevant sensitizations can unsettle the patient if no clear evaluation of clinical relevance is carried out. 

C. As low allergen-specific IgE concentrations (< 1 kU/L) cannot always be unambiguously detected by ImmunoCAP ISAC, this microarray is not able to definitely exclude an IgE-mediated sensitization. Particularly when total IgE concentrations are low, specific IgE values can be so low that they are only detectable by highly sensitive singleplex IgE procedures using an extremely low detection limit (≥ 0,1 kU/L). 

The most challenging task is probably the well-balanced evaluation of the multiplex test results without frightening the patients or keeping them in the dark about the consequences. Thus, the test and its interpretation should be carried out by specialists in the field of molecular allergy who are able to evaluate the test appropriately. Beyond that, the test is an excellent diagnostic tool that will be able to render valuable service to scientific questions and epidemiological studies. 

Currently, health insurance providers in Germany do not pay for this test so that the patients have to bear the costs. 

## Clinical questions when components are used 

In contrast to the skin prick testing or in-vitro diagnosis using extracts, diagnostic work-up with allergen components can answer additional questions:

- Sensitization against one or more primary species-specific major allergen?
- Reaction to (pollen) extracts exclusively based on sensitization against cross-reactive minor allergens?
- Is a suspected food allergy a reaction to high-risk, stable food allergens (e.g., storage proteins in vegetable food)?
- (Mild) reactions to food exclusively based on instable allergen components (e.g., members of the Bet v 1-homologous vegetable food)?
- Are primary, specific single components able to better differentiate suspected double sensitization (e.g., against insect venom of bee and wasp)?
- Can analytical sensitivity be improved by the respective allergen components when signals against allergen extracts are weakly positive (e.g., due to low total IgE)?

The challenge is that these questions can only be answered individually for each allergen component or allergen source. Thus, each allergen component available for diagnostic work-up needs to be investigated for clinical usefulness. The basic prerequisite for this is that diagnostic work-up with allergen components reflects sensitizations, the clinical relevance of which needs to be investigated by patient history. 

## Interpretation and presentation of results of studies on diagnostic work-up using components 

**Figure 3.**
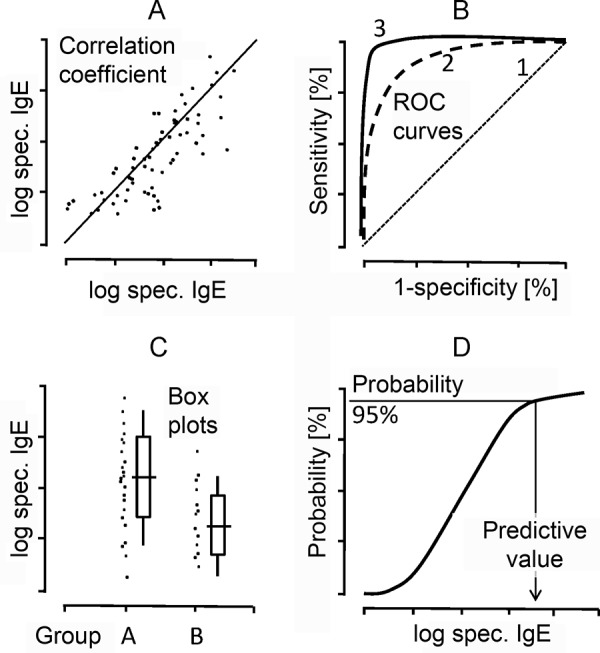
Typical evaluation of IgE laboratory results (e.g., using allergen molecules). A) Correlation of logarithmically distributed specific IgE concentrations (e.g., against a natural or recombinant allergen molecule). B) Diagnostic efficacy (ROC = receiver operating curves) when allergen (molecules) are used. C) Individual values, median values with 25% and 75% percentiles in between-group comparison. D) Limiting values for the prediction of clinical reactions (e.g., 95% probability of positive challenge test).

Clinical trials on diagnostic work-up using components frequently cover the same questions as have former studies on diagnostic work-up using extracts (Figure 3): 

*A) Are IgE values obtained with natural components comparable to those seen when recombinant single allergens are used? How far do values obtained by diagnostic work-up using components correlate with other clinical or diagnostic parameters?*

Reference values are frequently presented as single values and the corresponding rate of correlation (Figure 3A). Logarithmic presentation of the IgE values is desirable because IgE is not distributed linearly but logarithmically normal in the serum. 

*B) What diagnostic efficacy can be obtained with the single components?*

Diagnostic tests are evaluated using basic criteria like sensitivity and specificity. Furthermore, a positive predictive value (PPV) and a negative predictive value (NPV) are important criteria to assess the significance of laboratory results. These values depend on the prevalence of a disease and the composition of the cohort studied. The results are frequently presented using receiver operating characteristics (ROC) (Figure 3): 

A straight diagonal (line 1) would not have any diagnostic value due to lack of sensitivity and specificity. The optimal curve with an optimum of sensitivity and specificity would theoretically be situated rectangularly in the left corner of the diagram (see curve 3). 

*C) To what extent can specific IgE concentrations against components predict or exclude clinical reactions?*

Something that has always been wished for is the prediction of clinical reactions by quantitative measurement of specific IgE concentrations, e.g., against certain allergen components. Frequently, only group effects are obtained (Figure 3C, data set “groups A and B”) that do not allow an individual prediction due to their overlapping. The presentation of individual results with every single individual point would be desirable to be able to read the dispersion and individual variability directly. 

*D) To which probability can clinical reactions be predicted by diagnostic work-up using components?*

S-shaped curves are frequently used to illustrate to which probability (e.g., 95% or 98%) a clinical reaction can be expected at which IgE concentration, e.g., during food challenge testing (Figure 3D). However, the resulting (averaged) curves are suggestive and do not allow researchers to assess any individual variation. Furthermore, it has to be taken into account that these probability curves also depend on the prevalence of clinical diagnoses and the diagnostic findings in the center assessed. Thus, these study values cannot readily be translated to other clinical situations. 

Many examples also illustrate that the use of allergen components does not always allow a clear discrimination of the clinical reactions; frequently, group effects without an unambiguous individual predictive value are obtained. One of the reasons for this is that the tests using components is a mere proof of sensitization, which does not contain any information on clinical relevance per se. 

Despite these limitations, valuable studies, particularly on food allergens, were carried out in the past years (Table 4) that were supposed to answer the question as to whether allergen components can be used to diagnose a manifest allergy or tolerance. This also concerns the natural course of disease in infants and children, some of whom tolerate milk or egg in backed form after a while [24, 25]. In this context, results from the diagnosis with components would be welcome to be able to predict which children will tolerate certain foods better in the future. 

Similar questions can be asked in cases of inhalant allergies, i.e., in how far does the sensitization to certain components predict the success or failure of specific immunotherapy. So far, only retrospective results are available [26] but no prospective controlled studies; the exclusive sensitization to the birch pollen major allergen Bet v 1 is probably associated with a good effectiveness of SIT, while patients who are only or additionally sensitized to minor allergens like birch pollen profiling (rBet v 2), will benefit less from SIT [26]. The relevant allergens have been available for some time in the above-mentioned test procedures. Detection of IgE against marker allergens (Phl p 1, Phl p 5) hints towards a genuine grass pollen sensitization, which is probably the prerequisite for a successful SIT [26]. 

## Individual interpretation of diagnosis using components 

Positive signals in the form of determination of increased specific IgE antibodies can be evaluated with a qualitative (positive versus negative) or a quantitative approach. 

### High analytical sensitivity of modern quantitative IgE tests

Most modern quantitative measuring approaches are sensitive enough to also detect IgE below the formerly established threshold of 0.35 kU/L. Singleplex assays for specific IgE are so sensitive that sensitization is assumed when values above 0.1 kU/L are measured. However, the amount of total IgE has to be considered: 

A) Extremely low IgE – not unusual in non-atopics (e.g., with allergy against hymenoptera venom) – is usually associated with low allergen-specific (absolute) IgE values (also against components). 

B) When total IgE is strongly elevated – frequent in patients with atopic eczema –, not only numerous sensitizations, but also relatively strongly elevated specific IgE concentrations (also against components) can be expected. 

### Reliable quantification of specific IgE using allergen components

If the components have been chosen and bonded successfully by the manufacturer, specific IgE can be quantified in a more reliable way than would be possible using allergen extracts where some components might be under-represented. Trials using all available components of an allergen source frequently correlate well with the results obtained when the total extract is used. In other words: the sum of the IgE concentrations against all components is approximately equivalent to the result of specific IgE against the total extract. 

### Multiplex procedures: lower sensitivity and no standard curve for total IgE

The sensitivity of multiplex methods is limited in the range between 0.3 and 1 ISU (corresponding to IgE values of approximately 0.3 – 1 kU/L) and does not achieve the analytical sensitivity of high-quality singleplex assays. Furthermore, for this method, there is no standard curve calibrated against the WHO standard for total IgE (WHO IRP 75/502). Thus, only with the help of singleplex assays it is possible to relate specific IgE concentrations to the internationally standardized WHO units (IU/mL = kU/L) using a heterogeneous calibration method. 

### Concept of primary sensitization versus cross-reaction

**Figure 4.**
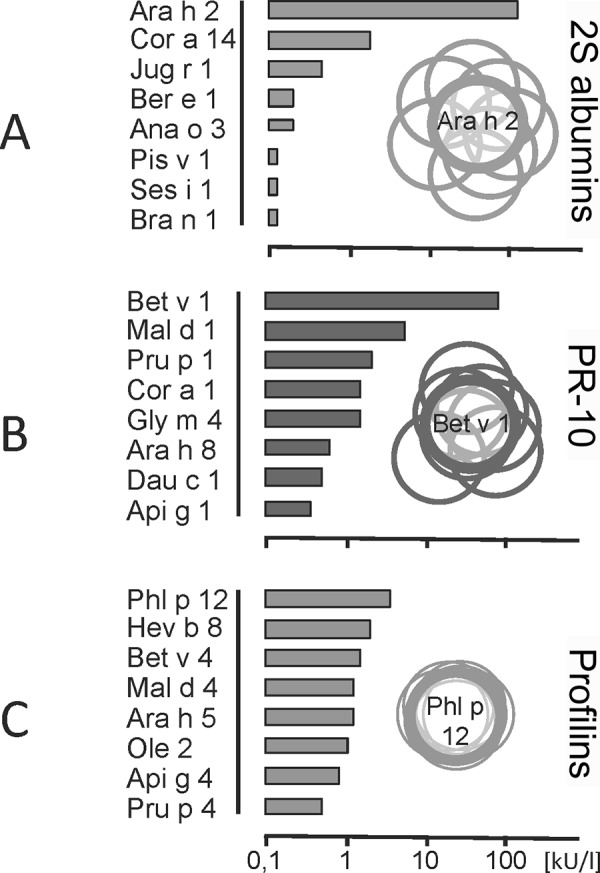
IgE concentrations against allergen molecules according to structural similarity within an allergen family. A: Variable, limited cross-reactions in 2S albumins (stable storage proteins in nuts, legumes, and seeds). B: Variable cross-reactions between Bet v 1-homologous food allergens. C: Pronounced cross-reactions due to strongly conserved similar structure of profilins (in pollen, latex, and food).

If the components show similarities (i.e., the allergens stem from the same, structurally similar allergen family), it has to be ascertained which was the primary sensitizer or if cross-reactions play a role. If the components were chosen correctly and their preparation was successful, the primary sensitizer can be identified roughly by the amount of the quantitatively assessed specific IgE concentrations (Figure 4). 

Probably, the highest value against a member of the protein family (e.g., storage proteins like 2S albumins) will represent the primary sensitizer, while the lower values possibly represent cross-reactive components. 

### Evaluation of clinical relevance

The central question is that of the clinical relevance of the specific IgE concentrations measured: 

As a rule of thumb, a positive specific IgE finding corresponds to a sensitization/cross-reaction that is only clinically relevant when corresponding symptoms are present. 

A negative specific IgE finding (against one allergen component) largely excludes an allergic sensitization/cross-reaction, but only if:

- total IgE is high enough,
- the allergen is intact, sufficiently available, and
- the analytical test sensitivity of the method of IgE determination has been optimized and is high enough.

Finally, only a physician, and not the test, can assess the clinical relevance of an allergic sensitization/cross-reaction, independently of the use of allergen extracts or components. 

**Figure 5. Figure5:**
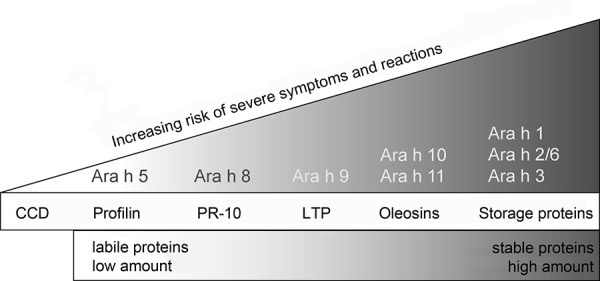
Allergy risk depending on stability and amount of allergen molecules in question. Ara h 1-11 = various peanut allergen molecules; CCD = cross-reactive carbohydrate determinants; LTP = stable lipid transfer proteins; PR-10 (pathogenesis-related protein family 10) = Bet v 1-homologues.

Thus, all diagnostic results – also those obtained by the use of allergen components – have to be evaluated taking into account the clinical context and the patient’s history. [Fig Figure5]

## Current state of analysis using single allergens 

In the following paragraphs, we present allergen molecules and their potential impact on IgE diagnosis, whereby this list does not claim to be complete. The examples given will illustrate that no global definition of the benefit is possible and that each allergen source has to be evaluated on its own. 

### Seasonal inhalant allergens

Single allergens for the detection of birch pollen or grass pollen sensitization have been available for some time. 

**Figure 6.**
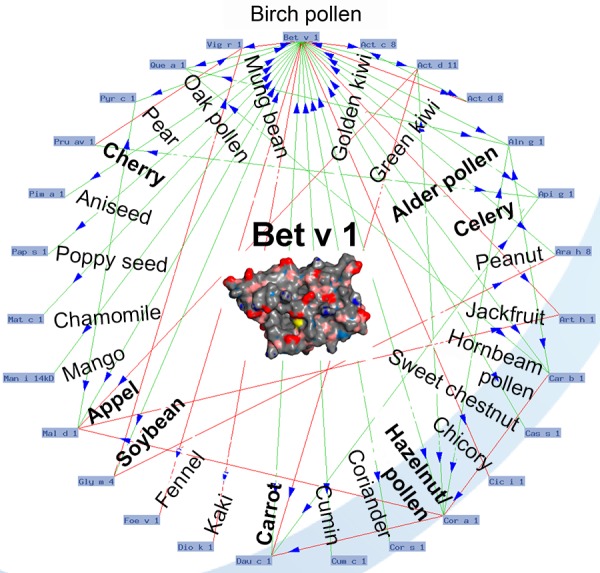
Allergome of the birch pollen major allergen Bet v 1 (3D structure in the center). Structural similarity (arrows) between Bet v 1 and similar PR-10 proteins in other tree pollen, fruits, and vegetables as a reason for birch pollen-associated cross-allergy, which is frequent in Central Europe (modified based on an O-ring of Bet v 1; dynamically generated in www.allergome.org; 3D-structure of Bet v 1 from www.ebi.ac.uk/pdbe-srv/view/entry/1btv/summary.html).

Strongly positive reactions to birch/hazel/alder pollen extracts (e.g., in prick or IgE testing) are mainly based on the Bet v 1-homologous major allergens; more than 95% of IgE reactivity against birch pollen derives from Bet v 1. This reactivity is the molecular basis for mainly mild oropharyngeal, sometimes also for threatening symptoms in patients with birch pollen allergy and pollen-associated food allergy. The parallel use of all Bet v 1-homologous food allergens (Figure 6) would not significantly improve IgE diagnosis because although they show sensitizations/cross-reactions, they do not allow conclusions on clinical relevance. 

The sensitization against grass pollen (e.g., timothy grass) entails a high degree of cross-reactivity with other allergen sources due to the fact that grass pollen allergens contain carbohydrate epitopes. In a primary, genuine sensitization against grass pollen, usually IgE against the recombinant major allergen rPhl p 1 is detected (in approximately 95% of all grass pollen sensitizations), often also sensitization against rPhl p 2, 5, and 11. Other available single allergens, rPhl p 12 and rPhl p7 (profilin and polcalcin), are considered to be strongly cross-reactive allergens, they are frequently not clinically relevant and tend to be responsible for multiple reactions against pollen extracts [18, 27]. 

Due to the lack of structural relationship, the major allergens of mugwort (Art v 1) and ragweed (Amb a 1) are suitable to differentiate IgE samples frequently cross-reacting to extracts in cases of herb pollen allergy. 

## Perennial inhalant allergens 

### Mold allergens

When mold allergy is suspected, the clinical picture of allergic bronchopulmonary aspergillosis (ABPA) has one characteristic feature, namely the exclusive sensitization against the intracellular *Aspergillus-fumigatus* antigens Asp f 4 and Asp f 6 [28]. Thus, IgE determinations, which are possible against 5 allergens of *Aspergillus fumigatus* (rAsp 1, 2, 3, 4 and 6), are used for the differentiation between allergic asthma and ABPA. 

### Animal dander

In this context, allergy against cats plays the most important role. rFel d 1, the major allergen, nFel d 2 (serum albumin), strongly cross-reactive with other mammal albumins (among others, Can f 3), and Fel d 4 (lipocalin, cross-reactive with Can f 6 and Equ c 1), are available for diagnosis. Currently, Fel d 5 (cat IgA) is being evaluated due to cross-reactions to meat caused by alpha-GAL sugar side chains (see below) [29]. Can f 1 (lipocalin) is considered the major allergen of the dog, but due to its partial cross-reactivity with Fel d 7 and other lipocalins it is not a reliable dog-specific marker. A further major allergen, somewhat less sensitive but highly specific for a genuine sensitization against dogs, is Can f 5 (arginine esterase), which is primarily only produced by male dogs. 

Apart from Fel d 1, no easy-to-use species-specific animal allergens that would allow a clear distinction of the corresponding sensitization against a certain animal have become apparent. 

### House dust mite

nDer p 1 and rDer p 2 as well as rDer p 10 are available as single allergens for allergologic in-vitro diagnosis. Der p 1, Der p 2, and the newly discovered Der p 23 [30] are considered the major allergens of the house dust mite *Dermatophagoides pteronyssinus.* Der p 10 is a tropomyosin that cross-reacts to structurally similar muscle proteins of other species, e.g., rPen a 1 of shrimps and Ani s 2 and Ani s 3 of roundworms (*Ascaris* species). Sensitization against mite major allergens is being discussed as a prerequisite for effective SIT [31]; the corresponding extracts are, at best, investigated for group 1 and 2 allergens (e.g., Der p 1 and 2) but not for other major allergens. Further house dust mite allergens might also be relevant and could be associated with asthma induction: Der p 5, 7, and 21 [32, 33]. 

### Latex allergens

Some latex allergens are available as single components for routine diagnostic work-up. Sensitizations against latex profilin (Hev b 8) is probably not associated with clinical symptoms, but the allergens Hev b 1, Hev b 3, Hev b 5, and Hev b 6 have been associated with latex allergy in health professionals and patients who underwent surgical interventions. The latex chitinase Hev b 11 (in addition to Hev b 8) is involved in latex-fruit syndrome [34]. 

## Cross-reactive carbohydrate determinants (CCD): 

The typical cross-reacting carbohydrate determinants (CCDs) are present in:

- pollen allergens,
- vegetable food,
- insects, mollusks, and
- some pathogenic helminthes.

Being epitopes in various allergen sources, they are responsible for many cross-reactions and thus are pan-allergens. Only rarely does their IgE reactivity appear to be of clinical relevance. 

Their glycan structures are composed of:

- N-acetylglucosamine,
- fucose (F),
- mannose (M), and
- xylose (X),

with IgE reactivity depending significantly on the presence of alpha-1,3-linked fucose (frequently) or xylose (rarely). Plant-based N-glycans show various steric constellations of the MMX, MMXF3, and MMXF3 type. A typical representative of MUXF3 is pineapple bromelain, a representative of MMXF is horseradish peroxidase. Both glycoproteins are considered to be screening allergens for the detection of IgE against CCD. MUXF3 is available as an isolated CCD without binding to a protein peptide. 

The typical CCDs (MMX, MUXF types) are used for diagnosis in IgE double positivity against bee and wasp venoms. In most of the cases, IgE against CCD is the reason for this double positivity in-vitro. 

In-vitro sensitization against latex is also frequently based on the IgE binding against CCD without corresponding symptoms after latex contact. On the other hand, IgE against the recombinant latex single allergens Hev b 1, 3, 5, and 6.01 are associated with clinical relevance. 

The determination of IgE against CCD can also be useful when the relevance of food IgE findings, e.g., against peanut, is unclear. Increased drinking of alcoholic beverages seems to raise IgE titers against CCD and thus IgE against plant-based foods. 

The carbohydrate structure in mammal tissue (except in primates, new world monkeys, and prosimians) is galactose alpha-1,3 (alpha-Gal). IgE antibodies against this epitope are associated with systemic allergic reactions (delayed anaphylaxis, predominantly urticaria, no asthma). IgE against alpha-Gal can be found in patients with allergies against offal and mammal meat [35] and can be determined by new laboratory reagents (laboratory number Ro307, art number 14-4997-10, ThermoFisher, Freiburg, Germany) and an immunoblot test (laboratory U. Jappe, Research Group for Clinical and Molecular Allergy, Research Center Borstel, Germany). 

## Allergens in hymenoptera venom 

For serologic laboratory diagnosis, the recombinant allergens Api m 1 (major allergen of bee venom) and Ves v 1 and Ves v 5 (phospholipase A1 and antigen 5, major allergen of wasp venom) are used. Due to the heterogeneity of sensitization patterns and the lack of single allergens for diagnostic work-up, particularly in the case of bee venom (Api m 3, Api m 4 and Api m 10 [36]), not all existing sensitizations against insect venom can be determined as of yet. Thus, IgE antibodies against the venom extracts and, when parallel positivity is present (double positivity against bee and wasp venom extracts), against the available single allergen components (see above) and CCD screening allergens are determined at the same time [35]. 

## Allergens in plant-based food 

### Storage proteins (in many allergen sources, e.g., peanut, soy, wheat)

Due to their high amount (relative portion of total protein) and their extremely high stability against cooking processes, pH, and enzymatic digestion, storage proteins are allergens that can cause threatening systemic reactions (Figure 5). Relevant allergen sources are legumes (e.g., peanut and soy), tree nuts (e.g., hazelnut), seeds, grains, and cereals. Some storage proteins are already available for routine diagnostic work-up: 

Sensitizations against the peanut major allergens, Ara h 1, Ara h 2, and Ara h 3 can be associated with systemic symptoms. In particular, increased IgE against the 2S-albumin Ara h 2 is a predictor for severe allergic reactions [37]. Cross-reactivity between the three major allergens is very high [38] so that the co-sensitization against all representatives can also be associated with severe allergy. Pollen-associated sensitizations with cross-reactions to profilin (Ara h 5) or the Bet v 1-homologous Ara h 8 trigger rather mild local reactions that are mainly limited to the mouth and pharynx (Figure 5). 

IgE against storage proteins of soybean (Gly m 5 and 6) are associated with severe reactions after ingestion of soy (particularly in children) [39]. 

Thanks to the storage proteins Cor a 9 and Cor a 14 (2S-albumin), it has recently become easier to distinguish between systemic reactions after the ingestion of hazelnuts and frequently mild birch pollen-associated, Cor a 1-related cross-reactions. The same is true for the walnut storage protein Jug r 1 (2S-albumin). 

A wheat storage protein, the best-known representative being Tri a 19 (omega-5-gliadin), is considered to trigger exercise-induced wheat allergy. 

### Lipid transfer proteins in fruits and vegetables

Lipid transfer proteins (LTP) in vegetable foods are also thermo- and acid-stable and thus markers of systemic reactions. It is not yet clear why most patients live in the Mediterranean area. A primary sensitization to LTP in the skin of peach (Pru p 3) is suspected, which is frequently used as a marker allergen for the cross-reactive protein family. While in Southern Europe LTPs are also important in baker’s asthma, peanut, hazelnut, pome fruit, and stone fruit allergies, this could only be shown for single cases in Northern Europe (study on baker’s asthma [40]). 

### Bet v 1 superfamily

Among the single allergens responsible for cross-reactions, the role of birch pollen major allergen (Bet v 1) as a reason for tree pollen-associated food allergy has been investigated best. 

Due to the thermolability and the digestion sensitivity of Bet v 1-homolgous allergens, the stone and pome fruits containing these allergens (including thick-skinned fruits like hazelnut) and vegetables mainly provoke only mild oropharyngeal symptoms if eaten raw, but are tolerated well when cooked. Exceptions are soy, carrot, and celery; their Bet v 1-homologous proteins can, if eaten raw, trigger severe allergic reactions. 

Figure 6 (Bet v 1-O ring, www.allergome.org) shows the Bet v 1-homologous allergens in vegetable foods that have been identified so far and their availability for routine diagnostic work-up. 

### Single allergens of various protein families illustrated through the example of the kiwi: Complex sensitization patterns, geographic differences, and diagnostic parameters

Kiwi allergy in all its variability is a good example to illustrate the current challenges of molecular allergy. In some parts of Europe, kiwi allergy is mainly associated with birch or grass pollen allergy, but it can also be part of the latex-fruit syndrome (12 – 39% of patients react to kiwi) and can also cause severe symptoms as a mono allergy [41]. 

The proportion of patients with kiwi allergy who show oropharyngeal symptoms varies between 66% and 72%, depending on the study, and is frequently associated with pollen allergy. 18 – 25% of patients with kiwi allergy develop systemic reactions (urticaria, gastrointestinal symptoms, asthma to the point of anaphylactic shock) after eating the fruit [41]. As skin tests using commercial extracts do not correspond well with the clinical symptoms, fresh fruits are used for prick testing. This is highly sensitive, but not very specific (31%) [41]. The low diagnostic sensitivity of IgE laboratory tests is based on the low concentration or the lack of kiwi allergens in commercially available extracts. The endogenous protease activity of a kiwi protein (actinidin, Act d 1) accounts for 50% of the total fruit protein and contributes to the fast depletion of allergenic proteins. IgE against Act d 1 and Act d 3 seems to be associated with severe reactions (Table 5) [41, 42]. 

In an important double-blind, placebo-controlled trial, 30 patients with kiwi allergy were challenged and the specific IgE against the available single allergens was determined [41]. Patients who were exclusively sensitized against kiwi frequently reacted with Act d 1. Patients with birch pollen allergy who did not suffer from kiwi allergy instead showed IgE against the kiwi glycoprotein Act d 3 and the Bet v 1-homologous Act d 8. A similar pattern was demonstrated for patients with pollen and latex allergy (without clinical reactions to kiwi). Obviously, IgE against kiwi glycoprotein, kiwi profilin, and kiwi Bet v 1-homolgues is not necessarily associated with clinical reactions. Thus, the latter are considered typical allergens of pollen-associated kiwi allergy whose symptoms are frequently limited to the oral cavity. Actinidin (Act d 1), which is important for mono allergy to kiwi without reactions to other fruits, is obviously associated with severe symptoms [41]. 

**Figure 7.**
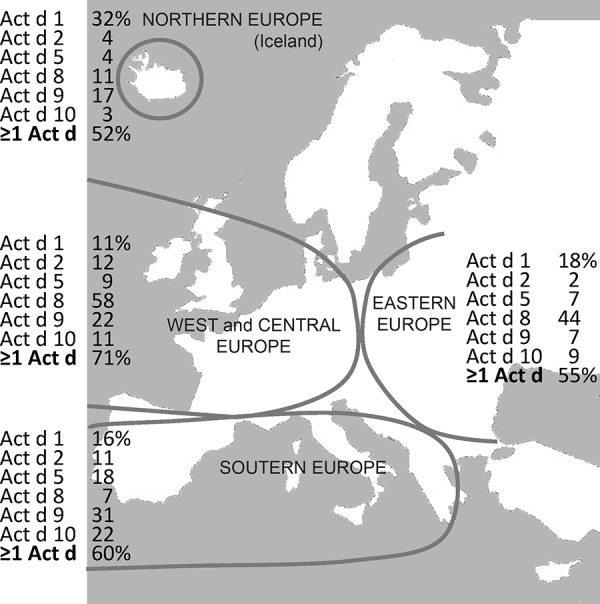
Sensitizations to kiwi (%) in various climate zones [43]. Kiwi allergens: Act d 1 (actinidin), Act d 2 (thaumatin-like), Act d 5 (kiwellin), Act d 8 (Bet v 1-homologue), Act d 9 (profilin), Act d 10 (nsLTP), ≥ 1 Act d = at least 1 kiwi component positive.

In Europe (Figure 7) [43], Bet v 1-associated Act d 8-mediated kiwi allergy with rather mild symptoms is mainly seen in Western and Central Europe, and less in Eastern Europe. Profilin (Act d 9) and LTP (Act d 10) sensitizations are more frequent in Southern Europe. Severe reactions are associated with Act d 1 sensitizations and have been described as mono allergies mainly in Iceland (the only representative of Northern Europe) [43]. 

Kiwi single allergens improve the quantitative test results of in-vitro diagnostic work-up (demonstrated in ImmunoCAP) and the diagnostic sensitivity as compared to the total extract. However, in some patients with proven kiwi allergen, no IgE against the components could yet be demonstrated [41]. This suggests that not all kiwi allergens have yet been detected. So far, no typical pattern for kiwi allergy in the context of latex-fruit syndrome could be detected using the currently available kiwi allergens. However, the sensitization patterns seem to vary according to geographic region, which seems to reflect concomitant inhalant allergies (climate-dependent exposure to birch or grass pollen) or the clinical severity (e.g., in mono allergies). 

## Animal food allergens 

While for plant-based allergen sources some marker allergens have become available [10], diagnostic work-up with single allergens of animal foods still only plays a minor role. 

### Cow’s milk and products made from it

The following allergens are currently known: 

Casein (nBos d 8, heat-stable); α-lactalbumin (nBos d 4, low heat stability); β-lactalbumin (nBos d 5, not heat-stable); bovine serum albumin (nBos d 6), and immunoglobulins (Bos d 7) [44]. 

However, analyses of the single allergens do not provide characteristic sensitization profiles of patients with cow’s milk allergy. Only reactions to beef in children with cow’s milk allergy seem to be based on specific sensitization against bovine serum albumin, now available as Bos d 6 [4^5^]. 

### Hen’s egg allergy

Known allergens are the thermo-stable ovomucoid (nGal d 1) as well as the less heat-stable allergens ovalbumin (nGal d 2), ovotransferrin (nGal d 3), and lysozyme (nGal d 4). All four allergens are major allergens in the egg white. Clinical reactions to the ingestion of hen’s egg are associated with the detection of IgE to albumen and ovomuoid (nGal d 1). Patients with persisting egg allergy have more IgE against nGal d 1 (ovomucoid) than against nGal d 2 (ovoalbumin) compared to children who develop tolerance [24, 44]. 

### Allergens in mammalian meat

Meat allergy can exist as a primary form or as a secondary form after inhalative sensitization against cat or dog components, for example. 

Before alpha-Gal was identified as an IgE epitope, serum albumins were considered to be important allergens in meat allergy (beef: Bos d 6). Further allergens are immunoglobulin G (beef: Bos d 7) with cross-reactivity to milk; myoglobulin, hemoglobin (beef: Bos d HG); a 81-kDa protein; and a 512-kDa phosphoglucomutase. 

Patients with a clear history for meat allergy usually show IgE against galactose-alpha1,3-galactose (alpha-Gal), expressed on mammalian cells and tissue. After Platts-Mills et al. published delayed systemic reactions after ingestion of meat when IgE against alpha-Gal was present, such observations are becoming more frequent in Europe as well [29, 35]. When “idiopathic anaphylaxis” is diagnosed, some of those cases might well be patients who react positively to meat due to an IgE-mediated sensitization against alpha-Gal. When IgE against alpha-Gal is detected and the patient’s history is inconclusive, challenge testing with meat should be carried out. Alpha-Gal is already available for routine diagnostic work-up or as an immunoblot test (see above, chapter “CCD”). 

### Crustacean allergens

Tropomyosin, an important muscle protein of all arthropods and other animals, is responsible for cross-reactions between mites (Der p 10) and seafood. They can cause heavy allergic reactions when ingested or inhaled: e.g., after shrimp (Pen a 1, Pen I 1), contamination with anisakis (Ani s 3), a parasite living in raw fish. For IgE diagnosis, extracts of the causative animal are also tested due to cross-reactive or species-specific allergens. At least one third of Central Europeans who are allergic to shrimps do not react to tropomyosin [46]. 

## Conclusion 

Molecular allergy is based on a) new concepts and b) increasing knowledge of the allergens responsible. Concepts for the differentiation of species-specific vs. cross-reactive marker allergens, risk and marker allergens can only be defined individually for each allergen source. 

Increased specific IgE concentrations against single allergens (allergen components) correspond to sensitizations that are only clinically relevant when corresponding symptoms are present. Thus, results of diagnostic work-up can only be interpreted together with the patient’s clinical information. 

## Acknowledgment 

We thank Doris Ruhland and Vera Wisliceny for the professional preparation of the manuscript and Ursula Meysel-Ecke and Sophie Wirth for the diligent correction. 

**Textbox 1.** Results and consequences of molecular allergen cloning (cf [3]).

Allergen definition: allergen nomenclature based on sequence
Discovery of new allergens
- Hierarchy of allergens from a common source
- Identification and understanding of cross-reactivities
- Grouping of gene families of major allergens
- Allergens for identification of 3D structure
- Geographic variants in allergen sequences
- Sequences suitable for peptide immunotherapy
- Recombinant proteins for diagnosis and immunotherapy
- Recombinant proteins for studies using defined and reproducible reagents
- Sequences for peptides for investigation of T-cell responses
- Species-specific allergens for epidemiological studies
- Alteration of genes towards hypoallergens
- Alteration of genes for improvement of immunotherapy

**Table 1.** Open-access internet sources/databases and information on molecular allergy.

**Website**	**Short characterization**
www.allergen.org	Official database of the IUIS Nomenclature Subcommittee with new and easier search function
www.allergenonline.org	Database on food allergens of the University of Nebraska, Lincoln, USA, Food Allergy Research and Resource Program (FARRP); carefully kept entries according to taxonomy of the allergen sources
www.allergome.org	Largest database on allergen molecules, initiated by the Italian allergist Adriano Mari and his team; partially entries on identified single allergens before their official naming
www.meduniwien.ac.at/allergens/allfam/	Database on allergen families (protein families) of the Medical University Vienna, Austria, Department of Pathophysiology and Allergy Research at the Center for Pathophysiology, Infectiology, and Immunology
www.allergyeducation-ma.com	Short, animated presentation of the manufacturer ThermoFisher (formerly Phadia)
www.phadia.com/de/Labors/Allergie/Webinar/Molekulare-Allergologie/	Dates for online seminars (webinars) and list of partially extensive brochures (PDF files for download) of the manufacturer ThermoFisher (formerly Phadia)

**Table 2.** Diagnostic work-up in cases of multiple pollen sensitization.

**Allergen source**	**Sensitization test**			
Tree pollen	+	+	+	+
Grass pollen	+	+	+	+
Herb pollen	+	+	+	+
				
Pollen pan-allergens				
Profilin*	-	+	-	+
Polcalcin*	-	-	+	+
	Real multi-sensitization	Probably “pseudo”-multi-sensitization due to cross-reaction to pollen pan-allergens profilin and/or polcalcin*		

*In Germany only available for determination of specific IgE in in-vitro testing; in Spain available as naturally purified prick test reagents (ALK-Abelló, Madrid) for the identification of cross-reactions to pollen pan-allergns.

**Table 3.** Selection of allergen components for diagnostic work-up using specific IgE.

		Providers in Germany:	Euroimmun	Dr. Fooke Laboratorien	Omega Diagnostics	Siemens Healthcare	ThermoFisher Phadia
		*Test systems:*	*Euroline*	*Allerg- o-liq*	*Allergozyme IgE*	*Immulite 2000*	*a) ImmunoCAP* ^5a ^ *b) ImmunoCAP ISAC* ^5b^
		Test concept and web information (cf legend)	Enzyme allergosorbent strip test^1^	Reverse enzyme allergosorbent strip test^2^	Enzyme allergosorbent strip test^3^	Chemilumenescence enzyme immunoassay with liquid-phase allergens^4^	a) Fluorescence enzyme allergosorbent test ^5a ^ b) Multiple fluorescence enzyme allergosorbent test^5b^
Allergen sources	Species	Allergen components					
Tree pollen	Birch	Bet v 1 (major all.)	n (SPAC*)	r (RT301)	n (x901)	n (A89)	r (t215)
		Bet v 2 (profilin)	n (SPAC)	r (RT302)	n (x907)	r (A127)	r (t216)
	Ash/Olive	Ole e 1 (major all.)		–	–	n (A482)	r (t224)
							
Grass pollen	Timothy grass	Phl p 1 (major all.)	n (SPAC)	r (RG601)	n (x903)	–	r (g205)
		Phl p 5 (major all.)	n (SPAC)	r (RG605)	n (x902)	–	r (g215)
		Phl p 7 (polcalcin)	n (SPAC)	r (RG607)	–	–	r (g210)
		Phl p 12 (profilin)	n (SPAC)	r (RG612)	–	–	r (g212)
							u.v.a.
Herb pollen	Mugwort	Art v 1 (major all.)	–	r (RW601)	–	n (A753)	n (w231)
	Ambrosia	Amb a 1 (major all.)	–	–	–	–	n (w230)
							
Rosaceae	Appel	Mal d 1 (Bet v 1H)	–	r (RF491)	–	r (A464)	r (f434)
	Peach	Pru p 3 (LTP)	–	–	–	n (A603)	r (f420)
							
Tree nuts	Hazelnut	Cor a 1 (Bet v 1H)	–	r (RF171)	–	–	r (f428)
		Cor a 9 (11S legumin)	–	–	–	–	r (f440)
		Cor a 14 (2S albumin)	–	–	–	–	r (f439)
							
Leguminosae	Peanut	Ara h 2 (2S albumin)	–	r (RF132)	–	–	r (f432)

r = recombinant component; n = natural component, purified from extracts, manufacturer-specific laboratory codes (in parentheses); Bet v 1H = Bet v 1-homologous PR10 protein; 2S albumin = storage protein; 11S legumin = storage protein; *SPAC = single purified allergen components, panel strip test (DP 3210-1601-1 E) with Bet v 4, Bet v 6,as well as extract of birch pollen and timothy grass pollen). Information on the test concepts: ^1^http://www.euroimmun.de/index.php?id=allergologie ^2^http://www.fooke-labs.de/downloads/flyer_allerg-o-liq_prinzip_email_2011-05.pdf ^3^http://static.omegadiagnostics.com.s3.amazonaws.com/product-downloads/ifu/Allergozyme_Spec._IgE_96T_-_36021000_-_DI02 0104_X_-_English.pdf ^4^http://healthcare.siemens.com/clinical-specialities/allergy/laboratorian-information ^5a^http://www.phadia.com/Laboratory/Allergy/Products/ImmunoCAP-Assays/ImmunoCAP-Specific-IgE/Test-Principle/ ^5b^http://www.phadia.com/Global/A%20Document%20Library/Allergy/Promotion%20Material/ImmunoCAP%20ISAC/ImmunoCAP- ISAC-Technical-brochure.pdf

**Table 4.** Examples of successful clinical validation of molecular allergy diagnostic work-up (plant-based allergen sources).

**Allergen source**	**Allergens**	**Comment**	**Reference**
Hazelnut	rCor 1.04, rCor a 2, rCor a 8, nCor a 9, rCor a 11	Clinical evaluation of the component-specific diagnosis in patients with hazelnut allergy from various regions (Denmark, Switzerland, Spain); diagnosis partially supported by controlled oral provocation, additional cohorts with pollen allergy and non-atopics; heterogeneous sensitization profiles depending on the region investigated.	[47]
Carrot	rDau c 1.0104, rDau c 1.0201, rDau c 4, rDau c IFR 1, rDau c IFR 2, rDau c Cyc	A) Clinical evaluation of 3 carrot allergens in patients whose carrot allergy had been confirmed by challenge testing compared to patients with birch pollen allergy without carrot allergy and non-atopic controls B) Clinical evaluation of the component-specific diagnosis in patients with hazelnut allergy from various regions (Denmark, Switzerland, Spain); diagnosis partially supported by controlled oral provocation, additional cohorts with pollen allergy and non-atopics; heterogeneous sensitization profiles depending on the region investigated.	[48, 49]
Cherry	rPru av 1, rPru av 3, rPru av 4	Clinical evaluation of component-specific diagnostic work-up in Central and South European (Spanish) patients with cherry allergy; diagnosis partially confirmed by controlled oral provocation, additional cohorts with pollen allergy and non-atopics; heterogeneous sensitization profiles depending on the region investigated and clear advantage of single allergens over extract-based diagnostic work-up (prick test; specific IgE with cherry extracts).	[50]
Celery	rApi g 1.01, rApi g 4, nApi g 5	Clinical evaluation of component-specific diagnostic work-up in patients with celery allergy; diagnosis confirmed by controlled oral provocation, additional cohorts with pollen allergy and non-atopics; clear advantage of single allergens over extract-based diagnostic work-up; nApi g 5-specific IgE is significantly directed against CCD.	[51, 52]

**Table 5.** Kiwi allergens identified so far.

**Allergens**	**Molecular weight (kDa)**	**Protein family**	**Cross-reactivity**
nAct d 1	27	Actinidin cystein protease	Unknown
nAcr d 2	24	Thaumatin-like protein	Unknown
nAct d 3	42	Glycoprotein with unknown function	Birch and grass pollen, latex
nAct d 4	11	Phytocystatin	Unknown Unknown
nAct d 5	26	Kiwellin	Unknown
Act d 6	18	Pectin methylesterase inhibitor	
Act d 7	50	Pectin methylesterase	Unknown
rAct d 8 #	17	Bet v 1-homologues (PR10)	Birch pollen
rAct d 9	14	Profilin	Birch and grass pollen, numerous plant-based foods, latex
Act d 11	17	Major latex protein	

Information based on www.allergome.org [41], ^#^available in modified form; Act d 1: marker molecule for primary kiwi allergy.
